# “I still don’t know how someone gets pregnant”: determinants of poor reproductive health among young female refugees in South Africa

**DOI:** 10.1186/s12905-023-02847-6

**Published:** 2024-01-03

**Authors:** Tamaryn L. Crankshaw, Jane Freedman, Victoria M. Mutambara, Yasmin Rajah

**Affiliations:** 1 Health Economics and HIV and AIDS Research Division (HEARD), University of KwaZulu-Natal, Durban, South Africa; 2 Centre for Sociological and Political Research (CRESPPA), Université Paris 8, Paris, France; 3 Refugee Social Services, Durban, South Africa

**Keywords:** Refugees, Young women, Reproductive health, Unintended pregnancy, Menstruation, Contraception, Abortion

## Abstract

**Background:**

Studies exploring the sexual and reproductive health (SRH) of refugee women have focused primarily on first generation refugees in humanitarian and crisis settings. There is a paucity of research exploring the reproductive health of girls and young women who are born to refugee parents in a host country or who have migrated with their parents at a very young age and who have since reached sexual maturity. We conducted a qualitative study which aimed to explore the reproductive health and rights’ needs and challenges amongst young refugee women in South Africa.

**Methods:**

The study was carried out in the city of eThekwini (Durban) in South Africa in 2021 and 2022. A total of 35 semi-structured, in person interviews were conducted amongst young refugee women between the ages of 18 and 24 years living in the city centre.

**Results:**

Twenty-five participants were 17 years or younger on arriving in South Africa, one of whom was born in South Africa. Eleven of these women had experienced one or more pregnancies while living in South Africa and all of these women had experienced at least one unintended pregnancy. Participants had poor reproductive health knowledge of the role of menstruation and how conception occurs. Economic, social, and legal insecurities intersected in complex ways as determinants of poor reproductive health outcomes. Despite availability, contraceptive use was poor and linked to lack of knowledge, myths and unwanted side effects. There were negative economic and social impacts for young refugee women experiencing early pregnancies irrespective of whether they were intended or not. Being unable to conceive or experiencing an unintended pregnancy negatively impacted sexual relationships which were entered primarily for material support. Desire for confidentiality shaped lack of access to legal termination of pregnancy in the public health sector.

**Conclusion:**

Participants experienced specific vulnerabilities resulting from their position as refugees despite length of stay in South Africa. It is important to better understand these specificities in the design of programmes and policies aimed at ensuring positive health outcomes for these young women. Peer education amongst refugee communities may be an important tool in the provision of culturally acceptable SRH education.

## Introduction

Whilst there is significant emerging literature on the sexual and reproductive health (SRH) challenges of female refugees [1–4], many of these studies have examined “reproductive health in crisis” [5] and predominantly explored the experiences of first generation women living in refugee/displacement camps in the context of humanitarian crises. Much of this scholarship pertains to maternal health and perinatal outcomes, sexual health linked to sexual and gender-based violence (SGBV), and access to concomitant care [6, 7]. Very little SRH-related research has focused on second or 1.5 generation [8, 9] refugee women and girls and those living in urban non-camp settings, particularly in Sub-Saharan Africa (SSA). These girls and young women, who are born to refugee parents in a host country (second generation), or who have migrated to this country with their parents at a very young age (1.5 generation) “may have entered a new cultural environment at a crucial time in their psychosexual development…(and may have) to contend with constructions of sexual and reproductive health from at least two cultures which may be at conflict on the matter” [10]. The challenges they face might be expected to be different to those of their mothers (the first generation) but also from those of young refugee women in camp settings through the influence of acculturation processes as a result of their settlement in urban areas. Finally, due to restrictive refugee laws and policies in destination countries they may find themselves in situations of political, economic and legal precarity, which have a negative impact on their sexual and reproductive health and rights (SRHR) [11].

While there is conflicting evidence around whether migrant women experience worse reproductive outcomes than do native women in host countries, attention has been drawn to the importance of the complexity of migration and the differential effects of integration/social participation on health [7, 12]. In their systematic review, for example, Gagnon and colleagues (2009) found that, overall, migrant women had as good or better perinatal outcomes than did native women but noted clear differences in outcomes linked to the geographical origin of migrants. They also noted the heterogeneity of definitions of migrants which limited their conclusions. Another systematic review examining pregnancy outcomes of native versus immigrant women in European countries found a positive link between naturalisation rates, levels of social support and pregnancy outcomes [12]. No definition for immigrant was provided in this review. A later review reported that asylum seekers had a higher incidence of unwanted pregnancies and induced abortion-to-live birth ratio compared to women in the host countries [13]. More recent research which examined the different categories of migrants highlighted the differences between asylum seekers and refugees and other groups of migrants. In a systematic review [14], women with asylum seeker and refugee status had poorer pregnancy experiences and outcomes than women in wider migrant populations or in the host country. The differences observed between asylum seekers and refugees, and other types of migrant populations underscores the importance of targeted research focusing on asylum seekers and refugees, for whom legal, political and economic conditions may create particular conditions of health vulnerability and difficulties in accessing health services [4, 11, 15].

SSA hosts over one fifth (26%) of the world’s refugees and many of these migrants are young people (20–24 years) [16]. Given the predominantly intra-regional migration flows on the continent, more attention needs to be paid to the intergenerational vulnerabilities these young people may experience, most especially as the migration generations attempt to settle and integrate into a host society [11]. Our research amongst young refugee women in South Africa who were either children native born of foreign parentage or young children, adolescents (10–19 years) or young adults (20–24 years) on arrival to South Africa suggests they encounter specific legal, economic and social conditions of precarity which impact on their experiences and their SRHR and knowledge. We explore here questions of reproductive health knowledge and challenges amongst these young women, and the implications of these for their reproductive health outcomes. We also suggest implications of our results for designing policies aimed at improving these young women’s reproductive health.

## Methodology

This paper is based on the research findings of a qualitative study which aimed to explore the reproductive health and rights needs and challenges amongst young refugee women in South Africa. The study was carried out in the eThekwini city centre (formerly known as Durban) in the province of KwaZulu-Natal, South Africa between September 2021 and May 2022. A total of 35 semi-structured, in person interviews were conducted amongst young refugee women between the ages of 18 and 24 years who were living in the city centre. Participants included second generation children of refugees born in South Africa, the generations of children of refugees who arrived in South Africa at a young age or during adolescence, and first generation primary refugees (see Table 1 for participant profiles). Nationalities of young women included Democratic Republic of Congo (n = 30)^1^, Burundi (n = 4) and Ghana (n = 1). A further 4 interviews were carried out amongst key informants from organisations that primarily support asylum seekers and refugees. The first group of participants were initially recruited through the Refugee Social Services, a non-profit organisation that primarily provides social services for refugees and asylum seekers in the KwaZulu-Natal province in which eThekwini is located, and thereafter via snowball sampling techniques. A second group of participants were recruited through the Usizo Lwethu clinic, a project under the Dennis Hurley Center to provide healthcare services to the marginalized communities in the province. Both organisations are located in the city centre.

**Table 1:** Participant information

Participant ID	Current Age	Age on Arrival	Date of Arrival	Nationality	Legal Status	Arrived with	Currently residing with
PID 1	20	7	2008	DRC	Asylum Permit	Mother	Mother
PID 2	23	17	2017	DRC	No papers	Boyfriend/husband	Boyfriend/husband
PID 3	21	Born in South Africa		DRC	Expired Refugee Status	Mother and father	Mother and father
PID 4	21	13	2013	DRC	Asylum Permit	Sister	Sister
PID 5	19	15	2018	DRC	No papers	Father	Originally Grandmother (now deceased) - now alone
PID 6	22	21	2020	DRC	No papers	Spouse	Alone (spouse left)
PID 7	20	12	2009	DRC	Expired Asylum status	Father	Alone
PID 8	20	9	2014	DRC	No papers	Mother	Mother
PID 9	22	11	2010	DRC	Asylum Permit	Mother (Separated from father during flight)	Mother
PID 10	19	3	2004	DRC	Asylum Permit	Mother and father	Mother (parents divorced)
PID 11	18	2	2004	DRC	Asylum Permit	Mother and father	Mother (parents divorced)
PID 12	24	11	2007	DRC	Asylum Permit	Sister’s friend	Sister
PID 13	21	3	2003	DRC	Refugee Status	Mother and father	Mother (parents divorced)
PID 14	22	18	2017	Burundi	Asylum Permit	Alone	Alone
PID 15	20	8	2012	Burundi	Refugee Status	Father and brother	Father
PID 16	21	16	2016	DRC	Asylum Permit	Sister	Mother
PID 17	23	18	2018	Burundi	Asylum Permit	Unknown woman	Spouse
PID 18	22	11	2009	Ghana	Asylum Permit	Mother and father	Mother and father
PID 19	23	6	2007	DRC	No papers	Mother	Alone
PID 20	23	13	2011	DRC	Asylum Permit	Aunt	Alone
PID 21	21	21	2021	Burundi	No papers	Alone	Alone (spouse left)
PID 22	23	15	2013	DRC	Asylum Permit	Mother and brother	Originally mother (now deceased) -now alone
PID 23	21	12	2009	DRC	No papers	Mother and father	Alone
PID 24	22	5	2006	DRC	No papers/status expired	Mother and father	Siblings
PID 25	22	18	2017	DRC	No papers	Sister	Sister
PID 26	24	17	2015	DRC	Asylum Permit	Alone	Alone
PID 27	22	9	2009	DRC	Refugee Status	Uncle	Spouse
PID 28	24	18	2015	DRC	Asylum Permit	Alone	Alone
PID 29	18	17	2021	DRC	No papers	Aunt	Aunt
PID 30	18	16	2020	DRC	No papers	Alone	Alone
PID 31	24	22	2020	DRC	No papers	Alone	Alone
PID 32	22	21	2020	DRC	No papers	Alone with 2 children (Spouse in DRC)	Alone with children
PID 33	24	22	2020	DRC	No papers	Alone	Alone
PID 34	23	18	2017	DRC	Asylum Permit	Aunt	Alone
PID 35	21	18	2019	Unknown (Born in Zambia to a Congolese mother and Zambian father)	No papers	Alone	Sisters

The interview guide explored young women’s refugee and family background, knowledge around their reproductive health, use of contraceptive methods, prior pregnancies and pregnancy outcomes and access to reproductive health services. Interviews were conducted in English or Swahili depending on participant preferences and all interviews took place in a private/confidential setting. An interpreter was used for interviews conducted in Swahili. The interviewer was a young Zimbabwean women in her early 30’s who also had a young child. Interviews were between 30 and 75 min long and were audio recorded. All participants provided verbal consent to participate in the interview and further verbal consent to have their interviews recorded. Obtaining verbal consent was a precautionary measure during the COVID-19 pandemic. Verbal consent was recorded at the beginning of the interview. Participants received ZAR100 to compensate them for refreshment and travel costs.

All interviews were transcribed and, through thematic analysis by the first three authors, collated and coded for themes and sub-themes. These were inductively derived through a coding process as laid out by Corbin and Strauss [17]. Ethics approval was obtained from the Humanities and Social Sciences Research Ethics Committee, University of KwaZulu-Natal (approval number: HSSREC/00002847/2021).

## Results

Twenty-five participants were 17 years or younger when they arrived in South Africa, one of whom was born in South Africa. Eleven of these women had since experienced one or more pregnancies while living in South Africa and all of these women had experienced at least one unintended pregnancy. Two participants reported becoming pregnant at age 15 and age 16 respectively. Below we discuss some of the determinants underlying these young women’s reproductive health outcomes.

### Misinformation around menarche and lack of knowledge about conception

There was a marked lack of reproductive health knowledge amongst many young women we interviewed, most strikingly seen through the lack of knowledge around how and when conception occurred. In a number of the interviews, the researcher needed to provide explanation as to how a woman may become pregnant. For example, when asked about the acceptability of pregnancies within her community, the following young woman, who had arrived in South Africa at 13 years of age replied:

Sorry, the question that you are asking me now about pregnancy is making me confused because I still don’t know how someone gets pregnant. (PID 8, 20 years old, Congolese)

The above participant had very limited engagement with the schooling system since she indicated she dropped out of school before the age of 16 years to assist her mother in financially supporting the household:

My mom was not the one who said: “No! You should stop going to school”. You could just see that things were not going well and sometimes you would come back after school and you would find her sitting in the house crying. So, there would be no food to eat and then sometimes there would be no money to pay for school transport. So, we decided that we will not be going to school and we will try and help her (PID 8, 20 years old, Congolese).

Other participants who had attended school referred to SRHR-related information they had received in class through ‘Life Orientation’, a subject which includes age-appropriate sexuality education as a compulsory component of the South African schooling curriculum. School is thus an important source of SRHR-related knowledge. But as the above quote illustrates, for many of the young refugee women, legal and economic insecurities relating to their refugee status, meant that they had limited and disrupted schooling.

Several young women reported not going to school because they did not have the correct legal documents. In one of these cases, the young woman told us she became pregnant when she was 15 years old. The father of the child was a man who was 15 years her senior.

(Becoming pregnant) was not my plan. My plan was to go to school, finish school, get a job, after that get married and then have children. At my age, I feel like I spoiled my life! (PID 5, 19 years old, Congolese)

Sexuality education has a key role to play in the education of young people but the importance of quality delivery of the curriculum was highlighted when some participants shared that while they had learned about how conception occurs in public schools, the timing and scope of content was variable:

Actually, we were learning about sex in school (in South Africa), but you know it was not about if you sleep with a man you will get pregnant, we were just talking about periods and we got that in (Life) Orientation [subject that includes sexuality education]. (PID 19, 23 years old, Congolese)

The following excerpt highlights how education on reproductive health issues can result in a positive experience for young women at menarche. A Burundian participant who had attended a Muslim boarding school from 10 years of age shared her experience of menarche:

“I was doing grade 6 and I was 13 years old. I wasn’t that shocked because just the day before we had just talked about it. You see we do have madrassa (religious school that teaches Islam) in boarding school and our *appa* (elder sister) was tackling that topic of menstruation and telling us how you take care of ourselves, and then luckily after discussing that topic, the next day I got my period at school” (PID 15, 20 years old, Burundian).

An additional source of information was female relatives. But in some cases the information provided by these older women was incorrect and anxiety inducing, designed more to scare them away from sexual relationships than to inform them about their reproductive health. Most participants indicated that when they began menstruating, they were taught by a female relative about how to take care of their personal hygiene as well as being told to “not let men touch them” or otherwise risk becoming pregnant, without any further elaboration on the significance of menarche and how pregnancy could occur:

Researcher: Okay, so after you told (your mother) about your period, did she, like have a talk with you and what was it all about?

Participant: Just like, don’t touch any guy. Don’t let any guy touch you, you will get pregnant! Those things. And it was with me every time that if a guy touched me I would get pregnant. So, if a guy touched me, I would just run home and tell my mom…when I got to grade nine, and they used to teach us those things...sex, intercourse, the guy’s penis enters into the vagina, and then that’s when I was like, “Oh, okay, this is how you get pregnant!” (PID13, 21 years old, Congolese).

Other participants shared very similar experiences:

So, she’ll be like, No, avoid boys whenever you have periods. Because I had many male friends around me. So, she would be like, “No! Tell them not to come when you are on your periods. If they touch you, you’re going to get pregnant.” And I am like, “Okay, fine.” I was actually believing what she was telling me until I grew older …I was actually growing with that idea of, if a man touches me I will be pregnant. I remember even during, like, my menstruation time, and men touched me I would panic thinking that I’m pregnant. (PID 9, 22 years, Congolese)

A Congolese participant explains some of the cultural silences around sex between Congolese parents and children:

In Congo, they have like…, I don’t know if there is Life Orientation in Congo, I don’t think so. Like Congolese people, they don’t want to tell children everything. They hide and keep things. Like, they think that, if they tell you something, you will go and try it and go and do such a bad thing. They don’t tell anything. Yeah. Like, as I grew up, my mom, the only thing that she told me was that: “Don’t play with men!” If men try to touch me, I will get pregnant. But we never sat and talked about all of these things directly (PID 22, 23 years old).

However, when the Ghanian participant recounted her experience when she first started to menstruate, she also echoed the lack of discussion with her parents around sex:

[My mother] just started saying that “Oh, now you can have a baby, so be careful.” She was like “Okay, it’s a good thing and now that you’re here, you’re becoming a woman.”… my parents don’t like to have the sex talk, I don’t know, they feel like they’ll push you into doing it. They didn’t actually talk about it. I don’t know, she didn’t go in deep with it but I knew what she meant. (PID18, 22 years old, Ghanian)

These silences mean there is a vacuum in knowledge for young women who then have to rely on others for the reproductive health information they require. Another Burundian participant shared that her husband was the one who provided her with information and advice on how to prevent pregnancy. While she had a positive experience in obtaining information from her husband, many others may not highlighting the importance of widely accessible information well before having a child. The participant told us that “the married ones usually have a better experience when it comes to protecting themselves (from unintended pregnancy) because they would normally have given birth. And when they go to the clinic or hospital that is where they are educated and informed about all these things that you can use to protect yourself” (PID18, 23 years old, Burundian).

Lack of knowledge around how conception occurs meant that some of these young women did not know when they, in fact, did become pregnant. The following participant, who had dropped out of school due to financial constraints, went to the clinic because she had stomach cramps and was vomiting. It was there that she discovered she was 2 months pregnant:

I only knew when I went to the clinic. Even though I went to the clinic, I didn’t believe what was going on. (PID 4, 21 years, Congolese)

Another young woman who also dropped out of school due to lack of financial support shared a similar experience, telling us that she only received information about conception and pregnancy when she went to hospital. She was 6 years old when she arrived in South Africa:

When I got pregnant, I didn’t understand how I got pregnant. I was actually 17 years and I gave birth when I turned 18 years. So, it was only at the hospital after I got pregnant where the nurse explained to me that yeah, it’s because of this and that. I didn’t understand that if you sleep with a man you will get pregnant. So, I got the answers in the hospital because my mom couldn’t tell me and my friends also didn’t tell me about it. (PID 19, 23 years old, Congolese)

As the above quotes suggest, the health sector is a key source of reproductive health information for young participants, albeit only for those who sought antenatal care. However, our data highlights a clear gap in provision of SRHR information for *primary* prevention of early and unintended pregnancies - before they occur:

Participant: Oh, yeah, just by the hospital, they were teaching us what to do when you do not want to get another pregnancy, you need to do this, this and that. So, since the time I was going for my pregnancy appointment, I would just tell them that they need to give me family planning after I give birth because I don’t want to get pregnant again.

Researcher: So you knew about contraception after you got pregnant?

Participant: Yes. I didn’t know that there was something that one could have to prevent pregnancy, if I knew about it before I would have used it and I wouldn’t have been pregnant. (PID20, 23 years old, Congolese)

Interestingly, one participant who was born in South Africa to refugee parents saw value in the provision of peer educators from the refugee communities to provide additional sexuality education which could potentially bridge some of the gaps in knowledge:

Yeah, yes, yes they can also provide intellectual people, who speak the Congolese languages and also they could just go by the community involving community groups. Maybe just picking a day or something, when they will have, like, ladies coming and just giving information to the younger women. They can even share about how things work in Congo and how to adapt now that they are here in South Africa. (PID 3, 21 years old, Congolese)

### Prevention and termination of unintended and unwanted pregnancies

Our research found poor contraceptive use amongst young participants. Contraception was reported to be freely available and proactively offered by health care providers in the public sector clinics, as well as accessible for a fee at pharmacies. Reasons for lack of contraceptive use included relationship factors, poor reproductive health knowledge, abstinence, unwanted side effects and fears around future fertility:

You see with Congolese they like giving information to other people, they always say that getting family planning is bad because maybe after four years you won’t be able to get pregnant and that usually makes a lot of the young women to not use family planning. So, some of the people do not use it, and even with me some of the people were telling me, why I was using it because they said that it was making me fat and my stomach was getting big. (PID 20, 23 years, Congolese)

Some woman preferred to rely on traditional methods contraception (e.g. periodic abstinence or rhythm method), as one young woman who experienced an unintended pregnancy explained:

(One of my friends) was just telling me that I have to protect myself and all that, and I had to go (to a large public sector Hospital) and it is free and all that, but then I wasn’t interested in all of that because I just thought that I wouldn’t get pregnant now. I trusted my body that much. I used to do all my calculations, like following my periods and everything and I knew that way. I was doing all of those things, so I was not expecting to get pregnant. (PID 23, 21 years, Congolese)

Preferred methods for preventing pregnancy amongst Congolese participants were barrier methods in the form of condoms and emergency contraception. A married Burundian participant shared that she had initially been using contraceptive pills but had become pregnant while on the pill. After the birth of her second child, the couple had agreed not to have any more children. She discussed contraception with her husband and now uses a 3-month injectable contraceptive (PID 17, 23 years old, Burundian).

Congolese participants acknowledged that if experiencing an unintended pregnancy out of marriage, women in their refugee community may seek to terminate the pregnancy to avoid any negative consequences.

I will say, yeah, in the refugee community probably most people would go for abortion because they do not want people to see that they are pregnant. Maybe they are living with their parents or the aunt and the aunt is so strict such that maybe if they find out that they are pregnant they will chase them out. (PID 1, 20 years old, Congolese)

While not all participants supported the idea of an abortion, most of the women we interviewed knew that Termination of Pregnancy (TOP) services were available in the formal and informal health sector.

I think in South Africa, it’s very easy to get abortion, even for (refugees). Abortion is like an option, you can decide if you want an abortion. (PID18, 22 years old, Ghanian)

Some women had personal experience or direct knowledge of other refugee women accessing formal TOP services in the public health sector while others also provided reasons why they and other refugee women may not access them, preferring to approach informal providers or self-induce using home remedies. A number of women alluded to the use of non conventional medical abortifacient in early pregnancy, including the mention of a deworming medication and a common antibiotic which has been linked with miscarriages in early pregnancy. Concerns around accessing public sector services included delays in getting prompt care, being in their second trimester of pregnancy and experiencing xenophobia or stigma around being a young refugee and pregnant.

They do go there (to the public sector clinic) but they always say that they are mistreated. One friend of mine who was pregnant and she always complained that she didn’t like going to the clinic for check ups because she was always mistreated because she was a foreigner and also because she was young. (PID10, 19 years old, Congolese)

Issues related to confidentiality, however, were the single biggest concern, especially in the case of young refugee women still living with a family member or due to reputation risk related to future marriageability.

People are doing it (having abortions), and they keep it (quiet) because it’s not something people are proud to do. People are not proud to say, “I aborted”. Because they might feel like, “I can share this with someone”, but then the reaction of the people wouldn’t let them, you know, narrate their problems. It’s very difficult because of the reaction, of how people would be viewing you. (PID 9, 22 years old, Congolese)

In this world, there’s no secrets. Even those nurses you’re going to… Okay, fine, you do abortion and then after that you get married to somebody. When you get pregnant, you go to the hospital and then the pregnancy it’s like four months or five months. You just go to the clinic, that’s necessary, and then the nurse will be there and they can just say ah, ‘You came to do abortion again?’ in front of that guy. Just tell me what will happen, it will bring me problems. Yeah, so I wouldn’t go for it. (PID 5, 19 years old, Congolese)

### Cross-cultural positionality and consequences of pregnancies

Despite the fact that many of the participants had lived in South Africa from an early age, they still made strong reference to the social norms of their country of origin, and of the impacts this had on their reproductive health decisions and challenges. For many, the idea of becoming pregnant outside of marriage was still unacceptable, as this young woman explains, attributing the norm to her Congolese culture:

Like for us Congolese they cannot, until you are married. Pregnancy is not normal unless you are married. If you’re married and you get pregnant it is not even an issue. But if you’re not, it’s a big problem, a big one, a big one! Like me, when I found out that I was pregnant, I did not tell my mom until it was like four months because I was scared because I knew that if I told her it was going to be a big, big issue. I think it’s only when you’re married, whether you’re 16 years, 17 years or 18 years. But if you’re already married, it’s normal to get pregnant. But if you’re not married yet, it’s always a problem. (PID 23, 21 years old, Congolese).

Interestingly, a Burundian participant spoke about the pressure on young Burundian women to conceive: “Once you get married, you must be pregnant” (PID15, 20 years old, Burundian). She added that many of these marriages and pregnancies were at a young age. She told us that “Muslim refugees get married quickly. They don’t see any problem with getting married at 18 years because they always say that you are ready and good to have a baby” (Ibid). This pressure to become pregnant was confirmed by a 21 year old Burundian participant whose husband had abandoned her because she had not become pregnant within the three months that they had been married. She told us he blamed her for being infertile. She told us that she did not have knowledge around how pregnancy occurred nor how pregnancy can be prevented. She expressed deep dismay around her fear of being infertile and felt that “something was wrong with her” (PID21, 21 years, Burundian). She believed that the 3 months that she had cohabited with her husband should have been sufficient time for conception to occur and felt that if she had conceived, her husband would have stayed and taken care of her.

Congolese participants who had become pregnant outside of marriage shared their experiences of being stigmatized within their refugee community. One young woman temporarily relocated to another province in South Africa to avoid the stigma. She told us:

It was very difficult for me and I even moved to go to Cape Town because I was very embarrassed and shy and people were talking about me. They would say: “You are very young”, this, this, “You want to go and give birth and you did not even finish school!” So, I left and went to Cape Town and I came back later. Like some people, they get married around 22 years, 23 years, and then you have to be at least married. I think that is why you see that a lot of Congolese, here and back home, never finish school because once you get pregnant, you have to drop out because if you continue going to school, they will laugh at you. Even where we are staying, if you are inside the house and you feel like you want to go and buy something, people will just be talking about you. (PID 20, 23 years old, Congolese)

Another Congolese participant shared her feelings of internalized stigma:

Like back home (in DRC), if you get pregnant at 16 years, 17 years, 18 years people will be like ah this girl, so young like this, you know the impression, and that’s how they are back home. Even here with my people, the ones that are here in South Africa. It’s always like that. When I got pregnant I was so, so, so embarrassed. I couldn’t even walk past a group of Congolese people because I was ashamed and I just thought they were going to gossip about me, and I had to hide. (PID 19, 23 years old, Congolese)

Social and religious norms around the acceptability of pregnancy only within marriage were also underpinned by key pragmatic, economic and social support considerations which cut across cultural and environmental contexts. Economic security is even more important for these young women given the precarity resulting from their refugee situation:

The right time for us is when you get married, and they have paid the lobola (traditional bride price) and everything. If you get pregnant then you know that they are going to take care of you. When you get pregnant before that it can be very difficult because the person might not even want to take care of you. (PID28, 24 years old, Congolese)

Indeed, as our data suggests, there were a number of negative economic and social impacts for young refugee women experiencing early pregnancies irrespective of whether they were intended or not. These included dropping out of school, losing employment and being told to leave their homes:

When I got pregnant, my sister chased me out. Yeah. I was even homeless! It’s like when you get pregnant, you have to go to that person who impregnated you. (PID 4, 21 years old, Congolese)

[My friend] was married and she was still doing grade 10. She stopped (school) and then she was staying with her husband. She told me she stayed at the husband’s place for maybe nine months or eight months then she stopped that marriage, but she has a baby now. Then she went back home and luckily, she had a good mind and she continued with her school and last year, she actually finished her matric and we are studying with her at [name of technikon]. (PID 15, 20 years old, Burundian)

The majority of participants were engaged in largely transactional sexual relationships which often took the form of arranged marriages, marriages of convenience or unmarried but cohabiting together. These sexual relationships were often rooted in women’s basic material needs and need for physical shelter.

You see we came here, and we do not have family, and somebody will be looking and saying where exactly can I stay with someone taking care of me. That thing is happening a lot. You question how you will be able to stay alone. If somebody looks like they are loving you, then you are going to go and stay with them and you end up getting pregnant. … that is when he is going to tell you that he is not even ready for marriage and having children and you will be left all alone. (PID 28, 24 years, Congolese)

As the above excerpt reflects, unintended pregnancies could destabilize these precarious sexual-economic exchange relationships which resulted in many young women finding themselves raising their children alone.

For me, it was not planned at all because at that time I was still young, I was 19 years and the life that I was living on that side was very hard, and at that time I didn’t even know whether I could get injection or pills to prevent so that I must not get pregnant. And the time that I got pregnant, I told the guy and then he said yeah you can keep it, if it is mine, you can keep it. But then after 3 months, he ran away, and I don’t even know where he went. In 2018, I heard that he is in Cape Town and I have never seen him again. No sign of him. (PID 26, 24 years old, Congolese)

## Discussion

Our research amongst young refugee women highlights a marked lack of SRHR knowledge and thus points to the important role of high quality comprehensive sexuality education (CSE), as an essential intervention towards good SRHR outcomes. CSE, when properly implemented, is a key strategy for reducing the risk of STIs, including HIV, and for preventing unintended pregnancies in young people [18–21] by equipping them with the necessary knowledge and skills to make informed decisions regarding sexual practices and contraception, amongst other key issues [18, 22–24]. Schools are a vitally important site for delivery of sexuality education and the South African Department of Basic Education has a long standing Life Skills Education Programme that is implemented through the Life Orientation Learning area in schools across all learner grades. The fact that over half participants spent their adolescent years in South Africa (and in the South African schooling system) means that poor reproductive health knowledge, including knowledge around the function of menstruation, points to the fact that, for our participants, schools have largely failed to mitigate for the lack of accurate information provided on these subjects by family members. Economic precarity and lack of legal status resulting in girls leaving school early due to financial issues and pressure to help parents by working, may also explain why some of the participants had seemingly received little or no CSE. However, poor SRHR knowledge may extend beyond our asylum seeker and refugee participants in the South African setting. A study exploring menstrual health and schooling experience amongst South African (non refugee) learners (16–22 years old) also reported girls being inadequately prepared for menarche, where learners expressed a number of other unmet SRHR information and support needs, including questions around pregnancy prevention, sex and contraceptive side effects [25]. The study also reported similar silences around sex and injunctions from female relatives at menarche to “stay away from boys” [25] as was found in our study where refugee adolescents were told to “not let men touch them”. Participant concerns around contraceptive side effects and myths and misconceptions around fertility were also common and have been widely reported in different female populations in sub-Saharan Africa resulting in a call for multi-layered and sectoral responses [26]. Lack of knowledge around widely available contraceptive options to prevent pregnancy need to be urgently addressed. While the evidence is unavoidably dated, child marriage is common in the DRC [27] and given the immediate pressures to establish fertility when married, there may be important gaps in contraceptive knowledge of young refugee women arriving in South Africa. The recommendation of one participant, referenced earlier, that specific culturally adapted out of school sexuality education be provided to young refugee women by trained peers from the same origin as their families is highly relevant. It also highlights the need to adapt CSE to be relevant to the experiences and backgrounds of all young people. Following a similar study in Sydney Australia, Botfield et al. [28] suggest that CSE could also be provided in tertiary settings to ensure that even those young people who do not attend school can benefit from this education. This suggestion is also important for the out of school South African setting where, as mentioned, several young women told us that they had left school early. The request for a knowledgeable person who is also rooted within the specific refugee community provides an important insight. The ability to maintain and continue embracing aspects of young refugee women’s respective cultures through peer education amongst refugee communities has the potential to act as a “bridge between communities and the health system …embedding SRH education in culturally acceptable programs (sic.), in consultation with community and religious leaders, (and) is a way of receiving community support on SRH issues” [29].

High rates of teenage pregnancies in the general population have also been well documented in the South African setting. Between 2017 and 2022, teenage pregnancies increased by 16.1% from 129 951 in 2017/18 to 152 292 in 2020/2 [30]. Amongst women of reproductive age, South Africa has the second highest prevalence of unintended pregnancy in sub-Saharan Africa [31]. However, experiences of pregnancy may be fundamentally different between the refugee and non refugee populations given the inherited and intersecting legal, economic and social insecurities they experience [4, 11]. The lack of family and State support for many of these young pregnant women, with those with precarious legal status unable to access State child benefit payments for example [11], contribute to these young refugee women’s specific vulnerabilities when they become pregnant or give birth. Further, although norms around pregnancy outside of marriage may have shifted in the new, more permissive sexual and reproductive rights context of South Africa with the Bill of Rights in the Constitution and as one of only 2 countries in the Southern and East African region that has legal provision for abortion on request, our findings revealed that stigma is still perceived and experienced and there remain many expectations around sexual relations and pregnancy that are rooted in participant’s more conservative “home” cultures. This led to lack of support for some participants who became pregnant outside of marriage and resulted in homelessness, increased economic precarity and/or over-dependence on a male partner. However, our research suggests nuances in community responses, for example, the social stigma around unintended pregnancies amongst Congolese participants seemed not because pregnancies were unintended per se but because they occurred *outside* of the economic and social safety net of marriage. It is in these ways, that the intergenerational impacts of forced migration resulting in fragmented or absent families and leading to a lack of family support networks increase insecurities for young refugee women and directly impact their SRH.

### Limitations

The majority of our participants were Congolese which means the perspectives of Congolese participants are unavoidably more dominant. However, where relevant, we have sought to include the voices of the remaining participants belonging to other nationalities. Given the small numbers of non Congolese participants we were unable to explore whether there were any differences in experiences between Congolese participants who are predominantly Christian and Burundian participants who were Muslim. This will be a valuable direction of enquiry in future SRH-related research amongst refugee women of differing religions but living in the same spaces. This was a qualitative study and therefore the results cannot be generalised beyond our sample.

## Conclusion

Our analysis of a group of young women from first, 1.5 and second generation refugee origin revealed important gaps in knowledge on reproductive health which, combined with socio-economic and legal precariousness resulting from their refugee status, led to negative reproductive health outcomes. Some of these issues, such as insufficient or limited sexuality education and early and unintended pregnancies, may be common amongst all young women in South Africa, but there are also specific vulnerabilities which result from their position as refugees despite length of stay in South Africa. In order to take these specificities into account, and to try and ensure more positive reproductive health outcomes for these young women, it is necessary to design programmes and policies which take into consideration both their socio-cultural inheritance and their socio-economic, legal and political situation in the host country.

## Data Availability

Due to the qualitative nature of the study, the data generated are not publicly available. However, further information about the data is available from the corresponding author upon reasonable request.

## Footnotes

1 PID 35 was born in Zambia to a Congolese mother and a Zambian father.

## Abbreviations

CSE: Comprehensive Sexuality Education
HIV: Human Immunodeficiency Virus
SRH: Sexual and Reproductive Health
SRHR: Sexual and Reproductive Health and Rights
STI: Sexually Transmitted Infections

## References

[1] Keygnaert I, Guieu A, Ooms G, Vettenburg N, Temmerman M, Roelens K (2014). Sexual and reproductive health of migrants: does the EU care?. Health Policy.

[2] Ivanova O, Rai M, Kemigisha E. A systematic review of sexual and Reproductive Health Knowledge, experiences and Access to services among Refugee, migrant and displaced girls and Young women in Africa. Int J Environ Res Public Health. 2018;15(8). 10.3390/ijerph15081583 PMC6121882 30049940

[3] Munyaneza Y, Mhlongo EM (2019). Challenges of women refugees in utilising reproductive health services in public health institutions in Durban, KwaZulu-Natal, South Africa. Health SA = SA Gesondheid.

[4] Freedman J, Crankshaw TL, Mutambara VM (2020). Sexual and reproductive health of asylum seeking and refugee women in South Africa: understanding the determinants of vulnerability. Sex Reproductive Health Matters.

[5] Austin J, Guy S, Lee-Jones L, McGinn T, Schlecht J (2008). Reproductive health: a right for refugees and internally displaced persons. Reprod Health Matters.

[6] Gagnon AJ, Merry L, Robinson C (2002). A systemic review of Refugee women’s Reproductive Health. Refuge.

[7] Gagnon AJ, Zimbeck M, Zeitlin J, Roam Collaboration (2009). Migration to western industrialised countries and perinatal health: a systematic review. Soc Sci Med.

[8] Rumbaut RG, Rose P (1976). The one-and-half generation: Crisis, commitment, identity. The dispossessed: an anatomy of exile.

[9] Rumbaut RG, editor. Ties that bind. Immigration and Immigrant Families Lawrence Erlbaum Associate; 1997.

[10] Dune T, Perz J, Mengesha Z, Ayika D (2017). Culture Clash? Investigating constructions of sexual and reproductive health from the perspective of 1.5 generation migrants in Australia using Q methodology. Reproductive Health.

[11] Crankshaw TL, Freedman J, Mutambara VM (2023). Intergenerational trajectories of inherited vulnerabilities amongst young women refugees in South Africa. Comp Migration Stud.

[12] Bollini P, Pampallona S, Wanner P, Kupelnick B (2009). Pregnancy outcome of migrant women and integration policy: a systematic review of the international literature. Soc Sci Med.

[13] Hadgkiss EJ, Renzaho AM (2014). The physical health status, service utilisation and barriers to accessing care for asylum seekers residing in the community: a systematic review of the literature. Aust Health Rev.

[14] Heslehurst N, Brown H, Pemu A, Coleman H, Rankin J (2018). Perinatal health outcomes and care among asylum seekers and refugees: a systematic review of systematic reviews. BMC Med.

[15] Mutambara VM, Crankshaw TL, Freedman J (2022). Assessing the impacts of COVID-19 on women refugees in South Africa. J Refugee Stud.

[16] United Nations. Migration Dynamics, Refugees and Internally Displaced Persons in Africa. https://www.un.org/en/academic-impact/migration-dynamics-refugees-and-internally-displaced-persons-africa (Accessed 14 July 2023) [.

[17] Corbin J, Strauss A (2008). Basics of qualitative research: techniques and procedures for developing grounded theory.

[18] UNESCO. The International Technical Guidance on Sexuality Education (ITGSE) (2009). An evidence-informed approach for schools.

[19] UNESCO (2014). Comprehensive Sexuality Education: the challenges and opportunities of Scaling-Up.

[20] UNFPA (2014). UNFPA operational Guidance for Comprehensive Sexuality Education: a focus on human rights and gender.

[21] WHO (2011). Guidelines on preventing early pregnancy and poor reproductive outcomes among adolescents in developing countries.

[22] Haberland N, Rogow D (2015). Sexuality education: emerging trends in evidence and practice. J Adolesc Health.

[23] Kirby DB, Coyle K, Alton F, Rolleri LA, Robin L. Reducing adolescent sexual risk a theoretical guide for developing and adapting curriculum-based Programs California. ETR Associates; 2011.

[24] Kirby DB, Laris BA, Rolleri LA (2007). Sex and HIV education programs: their impact on sexual behaviors of young people throughout the world. J Adolesc Health: Official Publication Soc Adolesc Med.

[25] Crankshaw TL, Strauss M, Gumede B (2020). Menstrual health management and schooling experience amongst female learners in Gauteng, South Africa: a mixed method study. Reproductive Health.

[26] Engelbert Bain L, Amu H, Enowbeyang Tarkang E (2021). Barriers and motivators of contraceptive use among young people in Sub-saharan Africa: a systematic review of qualitative studies. PLoS ONE.

[27] Male C, Wodon Q (2016). Basic Profile of Child Marriage in the Republic of Congo. Health, Nutrition and Population Knowledge brief.

[28] Botfield JR, Zwi AB, Rutherford A, Newman CE (2018). Learning about sex and relationships among migrant and refugee young people in Sydney, Australia:‘I never got the talk about the birds and the bees’. Sex Educ.

[29] Metusela C, Ussher J, Perz J, Hawkey A, Morrow M, Narchal R (2017). In my culture, we don’t know anything about that: sexual and Reproductive Health of migrant and Refugee women. Int J Behav Med.

[30] Barron P, Subedar H, Letsoko M, Makua M, Pillay Y (2022). Teenage births and pregnancies in South Africa, 2017–2021–a reflection of a troubled country: analysis of public sector data. South Afr Med J.

[31] Woldesenbet S, Kufa T, Lombard C, Manda S, Morof D, Cheyip M (2021). The prevalence of unintended pregnancy and its association with HIV status among pregnant women in South Africa, a national antenatal survey, 2019. Sci Rep.

